# Special Issue: Computational Analysis of RNA Structure and Function

**DOI:** 10.3390/genes10010055

**Published:** 2019-01-16

**Authors:** Jan Gorodkin

**Affiliations:** Center for Non-Coding RNA in Technology and Health, Department of Veterinary and Animal Sciences, University of Copenhagen, Grønnegårdsvej 3, 1870 Frederiksberg C, Denmark; gorodkin@rth.dk

RNA structure often plays a key role in determining the function of non-coding and coding transcripts. Whereas transcript abundance can help to identify transcripts of interest in or under some conditions, the RNA structure can help to explain how the transcript might function. Hence, identifying RNA structures encoded in genomic sequence is of great interest. Since RNA molecules are often folded intensively into structures, the functional important RNA structure is typically conserved and can be more reliably detected by considering the conservation of base pairs rather than the folding of a single sequence [1]. Although the map of base pairs make up the secondary structure of the RNA, it ignores the 3D or tertiary structure, which often can be obtained using a good secondary structure as starting point [2].

The detection of RNA secondary structures throughout multiple organisms can be achieved through different strategies: 1) excising the structure from sequence-based alignments, 2) aligning the predicted structure of individual sequences, or 3) simultaneously optimizing structure and alignment. This is reviewed in reference [3] by Pervouchine who discusses the challenges and further potential of the development of methods for detecting RNA structures which can have long range interactions in genomic sequences. 

In reference [4], Ivanov and Pervouchine study the type of long range RNA structure involved in regulating splicing of mutually exclusive exons [5]. Competing RNA structures consisting of semi-complementary elements that are located in introns up- and down-stream of an exon, can pair and thereby exclude an exon. Ivanov and Pervouchine propose that this mechanism results from duplications that have affected both exons and nearby base pairing introns. Their proposed model is based on several observations, including that there are increased selection of these elements flanking mutually exclusive exons. They also observed that these elements are more similar to each other than the sequences flanking other classes of exons.

Complementing the simultaneous fold and align strategy, which has a relatively high computational cost, using sequence-based alignments combined with a sliding window approach is faster. This strategy is used by Thiel et al. in [6] who, in contrast to previous human centered screens, use mouse centered multiple alignments to predict RNA structures in mouse and numerous other vertebrate genomes. Their findings are in agreement with previous screens and demonstrate that the RNA structure in the mouse is widespread and conserved in other vertebrates. Although Theil et al. control for boundaries of the pre-defined window sizes aimed to contain the local RNA structure in their screen, it is in itself relevant to accurately detect such boundaries. This is addressed by Sabarinathan in reference [7] who shows that the usage of multiple sequences enhances the detection compared to single sequences.

Another key challenge in detecting RNA structures is searching for those who change very dramatically in size and thereby structure over evolutionary time. A prime example is telomerase RNA, which is difficult to detect through both sequence and structure homology due to its variation. Waldi et al. [8] address this challenge in the Saccharomycetes genomes and discover 27 new structures by using a combination different sequence and structure search strategies.

High-throughput methods for RNA structure determination [9] provide information about single stranded and base paired positions. Although these do not point to the base pair partner, these are useful as a constraint in traditional programs for determining the secondary structure of an RNA molecule [10,11]. In contrast to folding, the data can be processed directly to search for structured motifs. In reference [12] Radecki and Aviran use a Hidden Markov Model framework to process such data. This has multiple advantages, including fast processing of large-scale data sets and fast comparison of structural patterns. 

Structural motifs are key components in the underlying architecture of the RNA structure [13]. In particular, recurrent motifs are prime candidates for carrying out specific functions [14]. Although detailed descriptions are useful, more coarse-grained descriptions are indeed useful as this captures the general architecture rather than smaller variations. Jain et al. [15] propose a graph based approach to study motifs and extract them from numerous existing structures. Their approach is also able to identify substructures in full size RNA molecules.

Many RNA molecules function by binding to proteins. The interactions between the RNA and protein give rise to the specific formation of molecular structures. Even though experimental data can provide the most reliable insight, computational analyses can be sufficient to point out relevant directions and to form hypotheses. In reference [16], Nithin et al. provide an overview of such tools with a focus on the 3D structure of RNA-protein complexes, their availability as web servers and/or standalone and their benchmarking.

Finally, some transcripts function through their ability to form circles and tens of thousands of circular RNAs have already been reported [17]. Detecting the circular potential on both coding and non-coding transcripts was addressed in reference [18] by Pan et al., who use a machine learning approach to predict this.
